# Editorial: Twenty Years After the Iowa Gambling Task: Rationality, Emotion, and Decision-Making

**DOI:** 10.3389/fpsyg.2017.02353

**Published:** 2018-01-25

**Authors:** Yao-Chu Chiu, Jong-Tsun Huang, Jeng-Ren Duann, Ching-Hung Lin

**Affiliations:** ^1^Department of Psychology, Soochow University, Taipei, Taiwan; ^2^Graduate Institute of Biomedical Sciences, China Medical University, Taichung, Taiwan; ^3^Institute of Cognitive Neuroscience, National Central University, Taoyuan, Taiwan; ^4^Department of Psychology, Kaohsiung Medical University, Kaohsiung, Taiwan; ^5^Research Center for Nonlinear Analysis and Optimization, Kaohsiung Medical University, Kaohsiung, Taiwan

**Keywords:** rationality, emotion, decision-making, Iowa Gambling Task, somatic marker hypothesis, ventromedial prefrontal cortex, expected value, gain-loss frequency

## Rationality and emotion in decision-making

Traditionally, the role of “emotion” has received little attention in research studies of decision-making (Finucane et al., 2000). However, 20 years ago, the “Somatic Marker Hypothesis” (SMH) proposed by the neuroscientist Antonio Damasio was introduced to explore decision-making under uncertainty (Bechara et al., 1994; Damasio, 1994). The SMH suggested that, under uncertain situations, second-level processing of the intact emotion system could facilitate rational decision-making in the long term. The core brain regions of the somatic marker (SM) system are believed to be located in the ventromedial prefrontal cortex (VMPFC) and orbitofrontal cortex, which integrate bodily signals from the peripheral to the central nervous system to create a response such as subjective feeling, and can also modulate and monitor decision-making (e.g., gut-feeling). The signals in the SM system can be regarded as a representation of certain positive or negative events or circumstances. In short, the intact SM system helps decision makers avoid disadvantageous choices or situations and instead consider advantageous choices or situations (Damasio, 1994, 1996).

Damasio and other notable neuroscientists also designed an examination tool referred to as the Iowa Gambling Task (IGT) that can be used to simulate dynamic real-life decision-making behavior as well as test the SMH (Bechara et al., 1994, 1997, 2000). This group of researchers evaluated VMPFC lesions using the IGT as a testing tool and recorded skin conductance responses (SCRs) to create an ideal experimental paradigm for exploring rationality and emotion in decision-making. The IGT has been used both as an indexical tool for studying the interaction between emotions and decision-making, and as a tool for clinical research and assessment (Bechara, 2007, 2016). The IGT has made a significant impact on cross-field research. In preparation for the publication of this special issue, “Iowa Gambling Task: 20 Years After,” we searched PubMed database using the phrase “Iowa Gambling Task” and found more than 400 IGT-related articles in 2012. Notably, the number of relevant articles has nearly doubled over the last 5 years to more than 800 in 2017. As numerous indices show, the IGT has provided a communal experimental platform for research in multiple fields that focus on issues related to emotions and decision-making.

## Validity issues with IGT investigations

The IGT is a gambling game that simulates a gain–loss experience in an uncertain environment. The gain–loss structure of the IGT utilizes four decks of cards marked A, B, C, and D. The selection of decks A and B results in a relatively large gain (US$100) in each trial and large losses (e.g., US$1250 in deck B) in some trials. The selection of decks C and D results in a small gain (US$50) in each trial and small losses (e.g., US$250 in deck D) in some trials. On average, selections from decks A and B over 10 trials will cause decision-makers lose US$250, and as such, these are defined as disadvantageous decks. Conversely, selections from decks C and D over 10 trials will cause decision-makers gain US$250, so these are defined as advantageous decks. The advantageous decks (C, D) provide small immediate gains in each trial, but the long-term outcome is positive; by contrast, the disadvantageous decks (A, B) provide large immediate gains in each trial, but the long-term outcome is negative (see Table 1, Bechara et al., 1994). Before playing the IGT, experimenters encourage participants to earn or avoid losing as much money as possible. However, at the start of the game, participants have insufficient information to guide them in making the right choice. They are also unaware of the internal gambling structure and the end result of the game. Theoretically, the participants are therefore situated in an uncertain environment. Furthermore, in order to gain the best outcome, participants would have to use their intuition based on their emotions determined by the SM system (Bechara et al., 1994, 1997, 2000).

**Table 1:** The first circle of 10 trials in the gain/loss structure of the IGT.

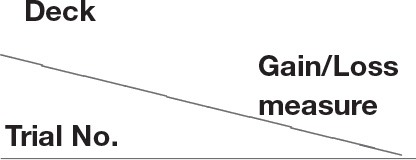	**A**		**B**		**C**		**D**	
	**Gain**	**Loss**	**Gain**	**Loss**	**Gain**	**Loss**	**Gain**	**Loss**
1	100	0	100	0	50	0	50	0
2	100	0	100	0	50	0	50	0
3	100	−150	100	0	50	−50	50	0
4	100	0	100	0	50	0	50	0
5	100	−300	100	0	50	−50	50	0
6	100	0	100	0	50	0	50	0
7	100	−200	100	0	50	−50	50	0
8	100	0	100	0	50	0	50	0
9	100	−250	100	−1,250	50	−50	50	0
10	100	−350	100	0	50	−50	50	−250
Net value	$–250		$–250		$+250		$+250	
Gain-loss frequency	10 gains		10 gains		10 gains		10 gains	
	5 losses		1 loss		5 losses		1 loss	

Note: The red marked the loss event; the blue marked the gain event; This table was sourced from Bechara et al. (1994).

Decision-makers receive gain/loss information after each round of card selection in IGT-related experiments. It is impossible to guess the internal gambling structure in advance, or to predict how to make the most money, but once the game is in progress, decision-makers gradually tend to prefer the good decks and avoid choosing the bad decks, potentially drawing upon physiological feedback. For example, their SCRs could be construed as an alarm signal that encourages the decision-maker to avoid selecting the bad decks before the cards are overturned. At the start of the game, participants are unable to differentiate between good or bad decks, but they exercise a “gut-feeling” in making selections for the IGT. This emotion thus influences decision-makers by guiding them to eventually choose only the good decks and thus obtain the best outcomes. Conversely, participants affected by VMPFC lesions are devoid of the SM system and are therefore unable to register gain/loss experience during the IGT. Therefore, VMPFC patients were unable to inhibit their preference for the bad decks, lost consecutively, and presented a shortsighted choice pattern (Bechara et al., 1997, 1999, 2000).

Nonetheless, some researchers have questioned the relevance of the IGT in testing the SMH (Dunn et al., 2006). Several other research teams have adopted the IGT to examine the SMH and have provided evidence that does not match the results obtained by Damasio's team. For instance, Tomb et al. (2002) have revealed that the amplitude of SCRs was unaffected by monetary and expected values (EVs) of cards during the IGT. Furthermore Maia and McClelland (2004, 2005) have found that decision-makers possess sufficient knowledge to detect the gambling structure during the early stages of the game, and as a consequence their processing is explicit, not implicit. In reply, Bechara et al. (2005) have emphasized that the SM signal does not just represent implicit processing. More specifically, healthy decision-makers mostly perform the IGT rationally and can be influenced by the SM system in either a covert or overt manner. In this manner, the original Iowa group has argued that the data reported by Maia and McClelland (2004) do not invalidate the SMH.

Furthermore, several researchers (Wilder et al., 1998; Fernie and Tunney, 2006; Lin et al., 2007; Chiu et al., 2008, 2012; Upton et al., 2012; Steingroever et al., 2013; Seeley et al., 2014) have discovered another critical issue that guides selection behavior during the IGT. All these authors have highlighted the importance of the number of gains or losses obtained, and not their expected value. The decision-makers in these studies considered choosing decks B and D due to the associated high-frequency gains and low-frequency losses, without considering the long-term outcome. Notably, the SMH has mostly based upon evidence gained by comparing the IGT performances and SCR responses of VMPFCs compared to healthy decision makers. An important point to note is that, based on the basic assumption of the SMH, healthy decision-makers should perform well and gradually approach the positive expected value choice in the IGT because of the alarm signals created by somatic markers and vice versa. However, empirical and modeling observations based on the prominent deck B (PDB) phenomenon and gain/loss frequency have clearly demonstrated a decision-maker's inability to consider long-term outcomes (or EV) in the IGT (Wilder et al., 1998; Ahn et al., 2008; Upton et al., 2012; Lin et al., 2013; Seeley et al., 2014; Worthy and Maddox, 2014; Lin et al.; Worthy et al.). Consequently, findings related to the PDB phenomenon and gain/loss frequency have clearly echoed the main points reported in previous literature concerning behavioral decision-making (Lichtenstein et al., 1969; Kahneman and Tversky, 1979; Tversky and Kahneman, 1981). In particular, the two viewpoints (SMH vs. behavioral decision) have separately represented foresighted and myopic viewpoints to interpret the decision-maker's behavior. Consequently, the two explanatory schemes were obviously controversial and incongruent in terms of understanding choice behavior under uncertainty.

If the frequency of gains or losses largely influences a participant's poor performance during the IGT, this finding not only belies the basic assumption of the SMH proposed by Damasio's team, but also calls into question whether the effects of gain/loss frequency could be observed in the data reported by Tomb et al. (2002) and Maia and McClelland (2004). It is particularly important to resolve the latter point because the findings of these two studies generally hinge upon the basic assumption of the SMH, in that the SM system assists the decision-maker in obtaining the best long-term outcome. If this basic assumption needs to be reexamined, then the arguments proposed in these two studies will also need to be reevaluated.

It is also important to highlight that an increasing number of studies are showing evidence that healthy participants exhibit myopic choice behavior similar to VMPFC patients (Caroselli et al., 2006). Furthermore, over the last 20 years, advancements in brain imaging technology have allowed such studies to include more clinical patients (Ernst et al., 2002; Fukui et al., 2005; Lin et al., 2008, 2015; Li et al., 2010), thus allowing the IGT to gain ground in becoming a useful tool for investigating the correlation between rationality, emotions, and decision-making.

In the meantime, modeling-related studies have also gradually enhanced our existing knowledge by shedding light on the cognitive processing of decision-makers while playing the IGT (Busemeyer and Stout, 2002; Ahn et al., 2008; Worthy et al., 2012; Steingroever et al., 2014). The papers we solicited for inclusion in this book also echo and expand on many of these issues.

## The special issue of “IGT: 20 years after”

In 2012, we started preparing this special publication, entitled “Iowa Gambling Task: 20 Years After,” and invited researchers from various fields related to IGT development from across the world to submit contributions. The proposed content includes reviews, prospective notes, as well as empirical, modeling, behavioral, and brain imaging studies. The chosen researchers were invited and peer-reviewed to present their knowledge and perspective on these issues. Based upon our suggestions, we expect the contributed papers to discuss the advancement of IGT-related issues. Papers were solicited from August 2012 till the end of 2015. A total of 24 papers were accepted that reflect the entire picture of IGT development over the past 20 years. These 24 papers can be divided into five categories as detailed below.

**Category I: Reviews:** (1) Must et al. review IGT and depression-related issues; (2) Brevers et al. review studies on IGT and gambling disorders; (3) Linnet provide a review of IGT in the context of dopamine and gambling disorders; (4) Cassotti et al. review IGT in relation to developmental studies; (5) Turnbull et al. consider IGT performance as the processing of emotion-based learning; (6) Overman and Pierce examine the effects of real plus virtual cards and additional trials; and (7) van den Bos et al. provide a global overview of rodent version of the IGT.

**Category II: Clinical examinations:** (1) Sallum et al. discuss the IGT and attention deficit hyperactivity disorder; (2) Xiao et al. combine the IGT and functional magnetic resonance imaging (fMRI) in order to investigate adolescent smoking behavior; (3) Singh describe the connection between sleep deprivation and IGT performance; and (4) de Oliveira Cardoso et al. provide a behavior-image study that investigates the correlation between frontal and cerebellar lesions and IGT performance.

**Category III: Model construction:** (1) Worthy et al. compare predictability between win-stay/lose-shift and Value-Plus-Preservation (VPP) models in the IGT; (2) Steingroever et al. validate the predictive power of the Prospect Valence Learning–Delta model; (3) Dai et al. provide an improved cognitive model for predicting IGT choice behavior; (4) Lin et al. refine a simplified model for estimating IGT performance; and (5) Ahn et al. compare three advanced IGT-related computational models.

**Category IV: Theoretical integration:** (1) Okdie et al. provide a statement on construal level theory for IGT-related performance; (2) Bull et al. consider sensitivity toward reward and punishment in healthy IGT participants; (3) Singh suggest a potential role for reward and punishment during the IGT; and (4) Singh consider the influence of sex-differences, handedness, and lateralization on IGT performance.

**Category V: Brain imaging technology:** (1) He et al. combine IGT and fMRI to investigate decisions involving unhealthy food; (2) Mapelli et al. utilize the IGT and event-related potentials (ERPs) to depict the behavioral performance and brain activation of patients with Parkinson's disease; (3) Tamburin et al. combine the IGT and ERPs to detect choice behavior and brain activation in patients with chronic lower-back pain; and (4) Fernie and Tunney describe a study on the correlation between SCRs and knowledge effects in the IGT.

The articles selected for inclusion in this special issue provide good coverage of neuroimaging modalities (ERP, fMRI, and SCR) used in previous IGT experiments. However, there might still be some room for a data-driven data analysis method (Mckeown et al., 2003) to relieve the limitation brought about by the fixed event structure used in a model-based method. After all, the brain responses to such a complex process might not always be time-locked to the event onset (Duann and Chiou, 2016).

## Conclusion

The 24 papers that form this new book are mostly consistent with IGT developmental issues over the past 20 years, such as the application of IGT in clinical scenarios, integrative investigations with combined brain imaging technology and the establishment of new models and theories. However, it is also necessary to continue global investigations and debate with regards to some existing and unresolved issues related to the IGT. For example: (1) What types of brain lesions (mental dysfunction) does the IGT truly measure? (2) Can SCRs be combined with the IGT to form a critical index of somatic markers? (3) Does the IGT measure ability for implicit or explicit learning? (4) Does EV or gain/loss frequency primarily guide decision-making behavior in the IGT? (5) Is it possible to devise a more sensitive data analysis method that can allocate more specific brain responses to the precise behaviors of IGT performance, such as the events of win, loss, and the switching of card decks? We recommend that future studies of IGT consider these questions seriously and provide in-depth investigations and discussions.

## Author contributions

Y-CC, C-HL, and J-TH discussed the main structure of this article. Y-CC and C-HL drafted the preliminary title, literature review, and chapter categorization, as well as the initial draft. J-RD and C-HL provided additional viewpoints for future development in the use of brain imaging for studying the IGT. J-TH and J-RD provided final refinements to this article.

### Conflict of interest statement

The authors declare that the research was conducted in the absence of any commercial or financial relationships that could be construed as a potential conflict of interest.
